# Environmental Equity through Negotiation: A Case Study on Urban Landfills and the Roma Community

**DOI:** 10.3390/ijerph13060591

**Published:** 2016-06-14

**Authors:** Ruxandra Mălina Petrescu-Mag, Dacinia Crina Petrescu, Ioan Gheorghe Oroian, Ovidiu Călin Safirescu, Nicoleta Bican-Brișan

**Affiliations:** 1 Faculty of Environmental Science and Engineering, Babes-Bolyai University, Fantanele Street, No. 30, Cluj-Napoca 400294, Romania; malina.petrescu@ubbcluj.ro (R.M.P.-M.); nicoleta.brisan@ubbcluj.ro (N.B.-B.); 2 Faculty of Business, Babes-Bolyai University, Horea Street, No. 7, Cluj-Napoca 400174, Romania; 3 Faculty of Agriculture, University of Agricultural Sciences and Veterinary Medicine Cluj-Napoca, 3-5 Manastur Street, Cluj-Napoca 400372, Romania; zoobiomag2004@yahoo.com (I.G.O.); calin.safirescu@usamvcluj.ro (O.C.S.)

**Keywords:** environmental equity, justice, landfill, Roma minority, negotiation, Pata Rât, Romania

## Abstract

The paper discusses the necessity to bring environmental equity within the Pata Rât Roma community in Northwest Romania, relying on the answers to three questions: “Does environmental equity exist in Pata Rât?”, “How can it be attained?”, and “To what extent can it be brought to the targeted people?” It was shown how a trio of factors tailors the destiny of Roma inhabitants: being a minority, their ethnicity, and the fact they are living on and off what society rejects and dumps—a landfill. The framing of the environmental equity concerns within a vision considering negotiation as the most adequate means to attain it is a novel approach. Further on, the results of the study can fuel win-win solutions in environmental equity. The information abstracted from a set of indicators, assessed through an evaluation matrix, represents a beneficial platform for future bottom-up decisions concerning landfill residents. Three action options were analyzed: on-site living opportunities—that resulted to be preferred, off-site living opportunities, and “Do nothing”. The analysis provides qualitative evidence that the evaluation of environmental equity is largely subjective, because of its complexity and specificity related to geographical, historical, cultural characteristics, and political interests.

## 1. Introduction

The Industrial Revolution, the spring of modern political ideologies, allowed mankind, for the first time in history, to improve its material life by wide-spreading welfare, but, at the same time, it caused irreversible natural environmental damages that has actually been threatening the very existence of humans. This paradox has fueled the emergence of a new ideology, namely environmentalism [1]. Environmentalism demands a fundamental philosophical orientation and a total political, economic, and social shift, as a reaction against the impasse created by people through merging anthropocentrism with the modernization brought by industrialization and by science in general. In this framework, equity becomes a *sine qua non* prerequisite for human development in a sustainable world and it should be pursued as a transversal objective, meaning that it has to be integrated into the definition and implementation of all policies. As part of this ideology, particularly in the Western world, the environmental movements of the late 1960s requested the acceptance of the idea that populations at the outskirts of society [2] were not only exposed to social inequities, but they bore a disproportionate share of negative environmental effects, without having significant involvement in the decisions causing them [3]. Environmentalism is rather an ideology than a political philosophy, although both are theoretical conceptualizations of politics [1], especially if considering its activist and materialistic character, strongly oriented towards masses. Consequently, it can be asserted that Environmental Justice (EJ) is one of the most evident manifestations of the ideological nature of environmentalism. A paramount achievement of the EJ movement is that it redefined environmental protection as a basic right and, thus, living in balanced environment represents a right, not a privilege reserved for a few who can escape and avoid a damaged environment and its consequences on them [4].

EJ is a controversial concept, an important bidder for research in social sciences, continuing to explore new directions and to elicit heated public debate worldwide. In this context, the present work aims to address a critical limitation in the EJ literature: the lack of investigation of a broad ethnic category (at Romania’s level), the Roma people, regarding their disproportionate exposure to adverse environmental conditions at the Pata Rât landfill (Cluj county, NW Romania). The main objective of the paper is to investigate the existence of environmental equity, how and to what extent it has the capacity to respond to the social, economic, and environmental challenges in the case of the Pata Rât municipal solid waste (MSW) landfill. Until now, EJ studies, dedicated mainly to the North American territory, have brought to the forefront of EJ analysis people of color or Latinos [5,6,7,8,9], scrutinized under different aspects. For example, Grineski *et al*. investigated various groups of Hispanics for EJ, looking at country-of-origin Hispanic subgroups of Cuban, Puerto Rican, Colombian, and Mexican, treating (quantitatively) differences within the Hispanic ethnic group in Miami [8]. However, it should not be disregarded that EJ themes have been explored, although in a smaller number of works, within the Central and Eastern European context, generating valuable EJ studies; they are dedicated mainly to Slovakia and Hungary and argue that race and class play significant roles in residential segregation and marginalization, with strong impact on the lack of EJ [10].

The framing of environmental equity concerns within a vision considering negotiation as the most adequate means to tackle and to attain it with long-term positive results for all stakeholders is a novel approach. The present paper brings to the forefront the idea that negotiation represents the best solution in resolving intergroup conflicts in this selected case study on the Pata Rât Roma community. Diversity in physical strengths, intelligence, education level, and cultural pattern among individuals create a whole range of differences, from those concerning basic human rights to quality of life, personal welfare, and power [11]. At the same time, people have similar needs, which can be grouped, if we follow Maslow’s theory, in physiological, safety, belonging, respect, and self-actualization [12]. The coexistence of various and limited resources and of more numerous needs generates a constant effort (from individual to societal level, targeted to the achievement of goals) that takes different forms, from pure cooperation to pure conflict. Usually, we are somewhere in between—we negotiate. The more parts are involved in a negotiation and the more complex the subject is, the more complicated the negotiation becomes. To ease the tasks of negotiators, the current work argues how the information abstracted from a set of indicators, analyzed through an evaluation matrix, represents a beneficial platform for future bottom-up decisions concerning the Pata Rât landfill Roma minority.

### 1.1. On the Way from Justice to Environmental Equity. Conceptual Framework

Justice is a contested and sometimes quite confusing concept. Nozick claims that justice requires absolute respect for property rights, even if this means great inequality between the wealthy and the poor [13]. Others consider that justice rests on solidarity within the community, fairness or traditional moral values [14]. Schlosberg explicitly presents EJ movements as the ones that “explore, represent, and demand justice—fair distribution, recognition, capabilities, and functioning—for communities as well as individuals” [15]. EJ is mainly concerned, according to Chakraborty *et al*., with its distributional dimension, with the disproportionate allocation of environmental “goods” and “bads”, where the burden of the “bads” is distributed especially within racial and ethnic minorities, lower income populations, and other vulnerable groups [16]. A definition that focuses mostly on the procedural side of EJ is that according to which EJ includes civil rights movement, normative goal of distributional fairness and community empowerment, as well as laws, regulations, and initiatives aiming to address disproportionate and adverse environmental conditions in ethnical groups and poor communities [5,17]. The same view is expressed by Bullard and Johnson: “Environmental justice is defined as the fair treatment and meaningful involvement of all people regardless of race, color, national origin, or income with respect to the development, implementation, and enforcement of environmental laws, regulations, and policies” [4]. The EJ is also considered a politically charged term which implies correction of an injustice imposed on a specific group of people [18], guaranteeing three basic rights: right to information, right to hearing, and right to proper compensation [19]. A supplementary conceptual clarification is appropriate in a discourse related to environmental or ecological justice: while the first is not usually preoccupied with the nature exterior to anthropic impacts, the latter focuses on doing justice to nature [15]. In most of the cases, environmental equity is seen as a broad concept, used to depict the disproportionate impacts—social, economic, environmental—of environment degradation. This approach folds with that of Nabalamba *et al*. and Walker, for whom environmental equity could be considered as the distributive component of justice, implying the equity in distribution of benefits and burdens and being the first pillar of EJ, while procedural justice, the second pillar of EJ, refers to equitable procedures through which justice is to be achieved [20,21]. Precisely, this procedural imprint of EJ targets the “access to and participation in decision-making processes and procedures that create environmental risks” [22]. The present paper focuses mainly on the distributional character of EJ (“who gets what” [2]), while its procedural dimension (“how it happened” [2]) is not taken under a large debate, considered to be too elaborate and overextended, which could deprive the analysis of a concise understanding. However, it cannot be overlooked that the use of negotiation may be regarded as belonging to the procedural justice, seen as participation in decision-making process. This delimitation in two dimensions of EJ (distributive and procedural) is not entirely clear and widely accepted in literature. For instance, Burton, when talking about distributional justice, refers to justice in distribution of goods, out of which two sub-categories emerge: process justice and outcome justice, therefore the procedural dimension is contained in the distributional one [23]; Cutter argues that environmental equity originates from three main sources of dissimilarity: social, generational, and procedural [24]. The vagueness could also be rooted in the “equity” concept, which, seen as a humanitarian issue, may embody considerations of fairness, justice [25,26], and equality [27]. Consequently, in the present paper, EJ and environmental equity should be taken as synonyms. Regardless of the robust and extended theoretical discussions on EJ, to which we have already referred to, mentions of equity, recognition, and participation are indissoluble parts of the universal language of EJ [28]. For many authors, a focus on distribution alone is not capable, anymore, to address the mechanisms of injustice, and may even reinforce them; therefore, EJ has to go beyond this distributional dimension to engage with that of “recognition” and thus, “justice-as-recognition” becomes a most promising conceptual approach to resolving tensions between social and ecological values [29]. The issue of recognition emerged by the fact that EJ activists often see themselves as outside the cultural mainstream, and, thus, their identities are devaluated. The EJ movement, then, turns to recognition as a key element of EJ [28]. In relation to the present research, a central factor for the Roma population is that of being recognized by public authorities to engage in decision making processes (in particular, the negotiations). In this context, the legal basis which ensures equal rights for minorities exists, but a positive discrimination is needed to guarantee a real participation of Roma people in issues of their concerns.

**Figure 1:**
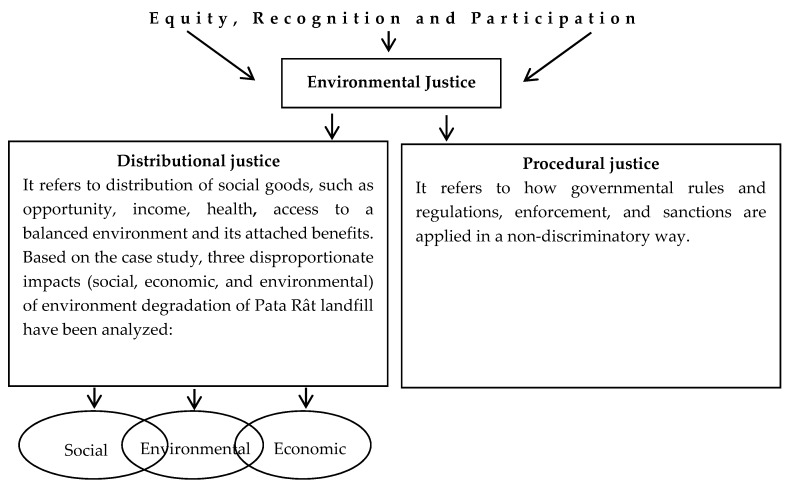
Conceptual framework for environmental justice (EJ) based on the under-analysis case.

Based on literature review [23,24,28] on justice and social justice models, a conceptual framework was designed in order to better place distributional and procedural components of EJ under the umbrella of equity, recognition, and participation (Figure 1).

Procedural justice, which reveals how governmental rules and regulations (soft and hard law instruments), enforcement and sanctions (international, European, and national) are applied in a non-discriminatory way, was not the central target of the present analysis, as it was already stated. Nevertheless, it is worth mentioning that case law on EJ is sparse and almost nonexistent at national level. Unlike the United States of America, where courts and society are constantly involved in the procedural exercise of environmental rights, in Romania, the prejudice caused by the adverse effects of economic activities on environment and human health was not recognized by the national magistrates until it had to be brought, finally, in front of the European Court of Human Rights (ECHR). There are only three cases against Romania—Băcilă, Tătar, and Brândușe—related to the right to a healthy environment and they were all successful. These are related to cyanide pollution (Tătar), pollution from a plant producing lead and zinc (Băcilă), and olfactory pollution in a prison (Brândușe). The first two are the most representative of the right to a balanced environment, which is why we included them in our discussion. The Tătar *vs*. Romania case, application no. 67021/01 delivered in 2009 [30], was rooted in a contamination generated by a technological process used by a company which exploited the Baia Mare gold mine. In fact, there was an ecological accident on 30 January 2000, when an amount of about 100,000 m^3^ of water polluted with cyanide was spilled into the Săsar river and from there in the Lăpuș and Tisa rivers. The applicants complained about the passivity of national authorities, who were deemed responsible for failing to take concrete measures to protect health and environment against pollution, invoking a violation of Article 8 of the European Convention on Human Rights (which was cited more often in cases of environmental harm than was the right to peaceful enjoyment of property, guaranteed by Article 1 of the Protocol 1 [31], and relying on the Court’s jurisprudence: López Ostra *vs*. Spain, 1994; Guerra *vs*. Italy, 1998; Moreno Gómez *vs*. Spain, 2004; Airey *vs*. Ireland, 1979; Hatton and others *vs*. UK, 2003). An infringement of the right to a healthy environment cannot be relied upon as such before ECHR, because it is not guaranteed *in terminis* by the Convention, and, therefore, the reference was made to Article 8, which regulated the right to have one’s privacy and property respected. Likewise the previous law suit, in the Băcilă *vs*. Romania case, application no. 19234/04 delivered in 2010 [32], the applicant based her action on Article 8, citing that environmental pollution caused her health damage (namely the effects of the pollution generated by a plant producing lead and zinc on the applicant’s health and living environment). The applicant did not denounce the functioning of the economic operator or the lack of information at the pollution level, but the fact that national authorities were unable to determine the economic agent to take measures to reduce contamination [33]. Consequently, in both cases, the Court stated that the operating conditions laid down by the Romanian authorities were inappropriate to preclude the possibility of serious harm. These lawsuits, Tătar *vs*. Romania and Bacilă *vs*. Romania, are two cases in which the ECHR reiterated that pollution can interfere with a person’s private and family life by harming his or her well-being. These matters of facts and their legal foundation may represent essential precedents for a possible Pata Rât case law. The Romanian waste problem received special attention, also from EU institutions, since the European Commission took action for the first time against Romania on the topic of waste, in September 2011. The reason was the country did not meet the 2010 deadline to transpose the EU Waste framework legislation (EU Waste Framework Directive 2008/98/EC) into national law [34]. Fortunately, the case was closed in the pre-contentious phase. Moreover, another two infringement procedures were launched in 2014 against Romania, because it failed to protect its citizens from the adverse effects of deficient waste management and because two tailings ponds from copper and zinc mines in Moldova Nouă have not been ecological rehabilitated, representing a major source of pollution, spreading toxic dust that posed significant risks to human health and the environment. At EU level, between 2010–2014, 208 environmental cases (preliminary rulings, infringements and other procedures) were brought in front of the Court of Justice of the European Union, from which 35 (17%) were on waste [35], indicating that waste has become a problematic subject of the European Union welfare society of the twenty first century, which requires an integrated approach solution (economic-environmental-social).

Cases of environmental injustice, brought in front of Court of Justice of the European Union, display serious discrepancies between the formal legal framework of human rights in relation with environment and their implementation [36]. Environmental conditions under which Roma minority resides and works is a measure of the success of the EU at recognizing and integrating marginalized communities [10].

### 1.2. Study Area

#### 1.2.1. Defining the Problematic Context of Roma People and of Pata Rât MSW Landfill

**Figure 2:**
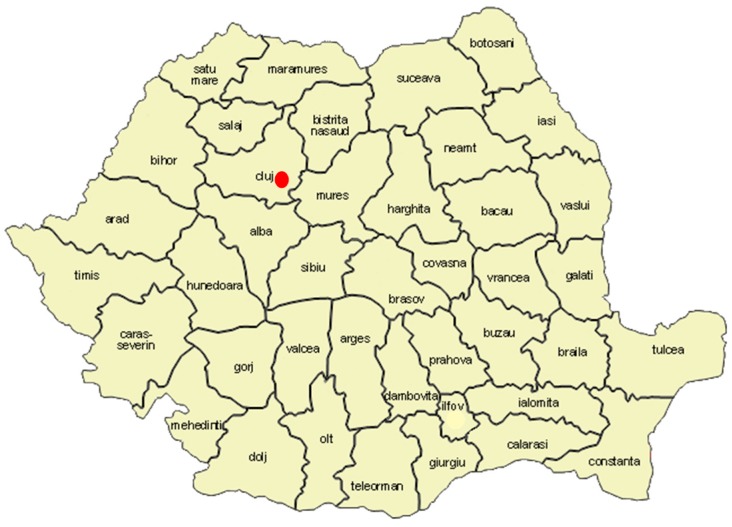
Location of the Pata Rât landfill (red dot) on Romania map [38].

Cluj county is situated in the North-West Development Region of Romania (North Transylvania; Figure 2) having a population of 691,106 inhabitants, out of which 22,525 (3%) people [37] declare themselves as Roma.

Pata Rât, counting over 2000 people, is located less than 18 kilometers from the Cluj-Napoca city center, being one of the most significant Roma communities. It consists of four settlements: Dallas, Cantonului Street, Colina Verde, and a village of shacks, next to the landfill, which was in fact, for twenty years (up to 2015), the municipality waste dump, representing the only source of livelihood for most of Pata Rât’s inhabitants. Romania has a total minority population of 11%, and the Roma population is the second largest minority (after Hungarians, which represent 60% of these), comprising 30% of all minority groups in Romania with 62,000 people (while the total population of the country is 20.1 million citizens) [39,40].

During the course of half a century, Pata Rât has become an area where people who had been experiencing difficulties in finding accommodation or workplaces in the context of mass-scale unemployment during the transition period and the perpetuation of negative prejudices against Roma minority, had started to reside and to increase in number [41]. Almost 40% of the residents were moved to Pata Rât from other places in Cluj by local authorities, as results of successive waves of evictions. Patterns of the Roma settlements vary from the urban “ghetto” type (Czech, Hungarian, or Western Slovak towns) to separate villages or shanty-towns (Eastern Slovakia, Romania, or Macedonia), but all mentioned types can be present in the same space [36]. An interesting approach, valid also in the Pata Rât case (from the perspective, for example, of evictions or low incomes, with consequences on the small number of owners, as shown in Table 1), is the one addressed by Filčák, who analyzes the unequal distribution of environmental benefits and risks, within a framework of entitlements – operationalized as land ownership and/or control [2]. He argues for the need to see entitlements not just as a part of the problem, but also as a part of the solution. Entitlements are seen as both driver of community development and as a brake in developing capabilities for those who are excluded from them. In general, in Central-East Europe, Roma settlements are perceived as “social pollution”, decreasing the well-being of those living in their close proximity, which is similar to the reaction to environmentally problematic activities expressed as the “not in my backyard” syndrome [2]. Similarly, the Pata Rât MSW landfill residents lead a particular existence, where poor environmental and economic vulnerable conditions (heavy metal contamination, poor housing conditions, work opportunities, and low income; Table 1), and social inequality (ethnic segregation, stigmatization, high delinquency, and lack of social assistance) [42] are combined into a desolate scenario of dehumanization.

The Cluj MSW landfill was opened in 1973 and designed to store 3.5 million tons of communal waste in an area of approximately 9 hectares, for a period of 30 years [45], but instead it was used 7 years more and supported 8 to 10 million tons of municipal and industrial waste, without preliminary separation or any pretreatment, out of which: 46% biological waste, 21% plastic, 17% paper and cardboard, 3% metals, 3% glass, and 10% other waste [46]. The current annual amount of garbage is high, raising to 160,000 tons (taking into account the Cluj population of 309,136 inhabitants, a student population of 57,595 persons that stay in the city at least 9 months per year, and an average waste production of 1.25 kg/day/person) [47]. The waste sector is mostly informal, being characterized as “universal, flexible, created by problems in the formal sector, composed by players that are invisible most of the time, not regulated by formal institutions, and difficult to assess in size and control” [48] and it can provide social capital, promote local economies, create jobs and contribute to environment protection [49]. In Cluj county, there is an intensive, active informal recycling sector with three components: street pickers that sort waste from waste bins or pilfer waste that is already sorted in special waste containers located on public domain; door to door collectors or itinerant waste buyers that go to households or even to economic agents and ask for waste materials as donation or for a low price; and landfill pickers who collect waste from the Pata Rât Municipal Landfill [50,51]. Since the end of 2010, two operators are authorized to manage the collection, transportation, and treatment of household waste generated in Cluj, for a period of at least 8 years. In the context of the lack of a mechanical sorting facility, a composting facility, or an ecological landfill, an Integrated Waste Management System for the whole county was needed and prepared by the public authorities using EU funds; the system will include: the construction of an ecological landfill, a sorting station and a mechanical-biological transfer station; the implementation of the separate collection of waste on five fractions (plastics/metals, glass, paper/cardboard, biodegradable waste and residual waste); and the closure of the improper Pata Rât landfill [52].

#### 1.2.2. Rational for Investigation of Para Rât Landfill Community

Pata Rât as a built environment, with its poor environmental conditions, lack of access to social security, and economic vulnerability, becomes a social-economic determinant [53]. In this context, vulnerability represents the risk of the Pata Rât Roma residents to being harmed by unforeseen events (environmental, economic, and social shocks) [54]. For the under-analysis case, it can be asserted that the damaged environmental and social conditions may induce economic vulnerability, becoming part of economic vulnerability and *vice versa*. Therefore, three components of the Pata Rât context—social (bearing the weaknesses of belonging to a minority and being disregarded), economic (with low income or lack of other resources), and environmental (suffering from high chronical contamination)—are interweaved and foster each other, creating a vicious circle, that is hard to escape from.

Huge diversity among needs, interests, perceptions, and values of Pata Rât residents, non-Roma community, and authorities imposes the use of a multilateral adjustment process of their demands and offers: the negotiation. Empirical evidence of previous top-down approaches designed to address environmental equity issues in Pata Rât confirms their inadequacy. Negotiation is understood as the basic means of getting what you want from others, though back-and-forth communication designed to reach an agreement when all sides have both common and conflicting interests [55]. Two negotiation types are widely known and used in practice, being the subject of numerous studies: win-lose and win-win, which belong to distributive and integrative negotiations, respectively. The first represents the process where what one party wins, the other one loses and where each party tries to maximize its share of a fixed-sum amount of benefits. In the second type, the win-win or integrative negotiation, partners attempt to explore their options with the purpose to enlarge the size of the common outcomes, without respect to the division of these. Open communication, willingness to share information, and mutual work to understand interests, values, and visions of each party and to be involved in a common effort to solve the problems are necessary for integrative negotiations [56]. The integrative negotiation is the only one adequate procedure to find a solution for the multilateral conflict regarding the Pata Rât community.

## 2. Experimental Section

To fulfill the research objective, the authors framed the environmental equity concerns in a conceptual framework (Figure 1), including negotiations as a means to achieve it. Negotiations need informational input, collected in this situation through an evaluation matrix. This was considered to be the most appropriate, because it can be applied in the case of subjective criteria, such as those specific for environment and social governance (loss of health, aesthetic benefits of environment, *etc*.). Additionally, obstacles in communication with Pata Rât inhabitants prevented the collection of large quantity of data, such as those required by a probabilistic survey. The evaluation matrix weights an idea (scenario/action option) in accordance to several factors or criteria, enabling the user to quickly identify the strengths and weaknesses of the options and to select the best one [57].

Within the research methodology, three action options were considered: on-site living opportunities, off-site living opportunities, and “do nothing”. The on-site option implies the creation of *in situ* improved living conditions for the Pata Rât residents: jobs, houses, water, sanitation, medical care, education, security, and a balanced natural environment (decontamination and prevention of future contamination). The off-site option consists of sending the residents from the village of shacks, located next to the landfill (and not all the people from Pata Rât) to better conditions, namely to the city, re-locating and integrating them into the municipal community, and not all of them together, but spread on 3–5 location points in/around the city; they would receive similar living condition to those from the “on-site” option. The “do nothing” option represents the continuation of the present status, without public or private intervention. The criteria for the evaluation of the action options were identified through a focus group session with twelve participants, three from each of the groups considered important for the study: local administration authorities, non-Roma city residents, NGOs, and Pata Rât (Roma) residents. The participation in the study was voluntary, the target group was formed based on convenience criteria, and the acceptance rate was 30%: out of 40 formulated requests to participate, 12 people accepted (one after another, 10 representatives of local authorities were asked to participate until, finally, three of them accepted; similarly, 10 city residents were asked to participate until three of them accepted; nine NGO representatives were requested to get involved until three of them agreed; and 11 Pata Rât residents were asked to participate until three of them accepted). The reduced level of willingness to get involved was based on motives such as lack of financial incentives, time, and interest in the topic. At their turn, researchers were limited by budget and time constraints to continue the selection of participants beyond the number of 12 participants (long delay in receiving answers and in finding a mutually accepted meeting timeframe). At the beginning of the focus group, the objective of the meeting, the research context (objective, concept of equity, the three action options, *etc*.) were briefly explained and all participants’ questions were clarified. Next, they were requested to come up with ideas about possible criteria for the evaluation of the action options. The results were noted by a member of the research team. The focus group generated a large list of criteria (19) that could be used for environmental equity assessment. A break was taken, during which researchers created and printed a table with the criteria that emerged during the discussions, with criteria arranged in the lines and importance levels in the columns, from 1 to 10. Level 1 represented the lowest importance and 10, the highest. After the break, each stakeholder received a copy of the table and he/she was requested to evaluate the importance of each criterion (by marking one level for each criterion) for the process of judging the efficiency of each action. This was done in writing, individually, without consultation among each other, and avoiding reciprocal influences. Focus group members who were not able to fill in the table by themselves were assisted by the researchers. Another break was taken and the criteria with the highest scores (16 out of 19) were retained by researchers for the subsequent stage and organized on three sections, social, economic, and environmental; the social one was structured according to Maslow’s groups of human needs (Table 2, first column). The same twelve participants received a printed table (with criteria on lines and action options on columns) to evaluate each criterion on a scale from 1 to 10 (with 1—the lowest level and 10—the highest) in relation to its capacity to be fulfilled within each specific action option. They had to fill in a number in each column for each line. The evaluation was made individually, without influence on each other and with help from organizers, when needed. In the case of five questions, the results were re-coded (reversely, 1 became 10, 2 became 9, and so on) by researchers (as mentioned in the footnote of Table 2) during the analysis process. The effects of each action option were judged on a time span of 5 to 10 years, because less than 5 years does not allow significant changes and more than 10 years is too far away in time, making estimations difficult. Each action option was explained in detail. Anonymity of the answers was granted to ensure higher objectivity.

All subjects gave their informed consent for participation in the study. The study was conducted in accordance with the principles included in the Declaration of Helsinki (1964, with subsequent modifications).

## 3. Results

The negotiation process will be fueled by the information supplied by the results of the evaluation matrix, which was judged to be the most appropriate for the present context, mainly due to its suitability to subjective criteria and no need of very large input data.

The results in the evaluation matrix indicate that physiological needs are considered by all stakeholders as the most important criterion to be used when an action option is analyzed (10 points received from each group), while the least important came out to be: for the authorities—“They are treated as equals by the people from their community/outside community”; for the non-Roma city residents—“They are treated as equals by the people from their community/outside community” and “They don’t contribute any longer to environment protection through waste selection”; for the NGOs—“They don’t contribute any longer to environment protection through waste selection”; and for Pata Rât residents—“They are a threat to the health of the population of the city—through their lack of hygiene, diseases, and bad habits” and “They don’t contribute any longer to environment protection through waste selection”. Thus, while for the authorities and the majority of the population the equal treatment is negligible in projection of public actions, for the Roma people, the issue of themselves as a threat to the other is irrelevant. These statements picture, once again, the existence of different priorities for each group, the clash of their interests, and they underline the necessity of a negotiation process to harmonize major gaps and to make environmental equity possible. This snapshot on stakeholders’ mentality indicates that major information and reparatory and prevention efforts are to be deployed over a long period of time in order to change behaviors, values, and beliefs deeply rooted in the consciousness of all stakeholder groups.

Among the three action options, the winner is to bring proper living conditions to Pata Rât waste dump (the “on-site” option), implying that this would have the best chances to be accepted as a negotiation outcome by all stakeholders.

From a practical perspective, in the negotiation process, the type of information provided by the present evaluation matrix becomes raw material on which reciprocal understanding of participants’ profile—interests, values, beliefs—and bargain tactics can be shaped to achieve a win-win solution for environmental equity.

## 4. Discussions. Contextualization of the Results in the Frame of Environmental Justice

### 4.1. The intercession of Negotiation in Supporting Environmental Equity

Negotiations among stakeholders are required in order to create and implement effective solutions that would lead to environmental equity. Due to the complexity and dynamism of the problem, it cannot be solved by unilateral and ready-made solution, but through integrative (win-win) negotiations among parties with similar power. The negotiation power (understood as negotiator’s capacity to direct negotiation towards its objectives) is influenced by many factors, from the mere capacity to be present at the negotiation table to the active involvement in each stage—pre-negotiation (information on the partners, subjects, exchange of preliminary information, *etc*.), negotiation itself (bargaining), and post-negotiation (implementation of the agreement and maintenance of the relationship with the other negotiation partners) [58].

Unfortunately, balanced negotiation power and integrative negotiations are very difficult to achieve in the present case, as a consequence of the asymmetric access to information, advantageous political power of one side, significantly higher economic power and better networking of some partners. The weakest partner is always the one representing Pata Rât residents. As Okereke observed, despite the egalitarian notions of justice in environmental agreements, strategies, and action plans, core policies are blocked in market-based neoliberal interpretations of justice, loyal to the *status quo* [59]. In a neoliberal era, approaching state with hope and faith, seeing it as a trustful partner and preaching its hegemony in terms of creating EJ, reflect the course of action adopted so far, one that failed and betrayed its activists, perpetuating institutional racism, and thus, imposing a rethinking of the attitude towards the state [60]. Similarly to the reality analyzed by Pulido *et al*. in U.S. [60], the need for a radicalization of EJ movement is present in Romania, too, due to the infiltration of capitalist interests in the public bodies responsible with EJ. Negotiations which have an extended environmental side are some of the most complex (being almost always multilateral) and difficult to resolve, and, to complicate the situation even more, they cannot be separated by social and economic aspects; they are time-consuming, need supplementary creativity, information, and they are usually preceded by extensive scientific fact-finding [61].

Intervention measures to decompress the forces that maintain disequilibrium are required to set the stage for the future negotiation process. One possible tool, designed to act within the preliminary phase of negotiation and also during the bargain stage, is information regarding the evaluations of the environmental equity by the stakeholders. Data and knowledge are crucial because they help participants to become more aware of their own positions and interests and to include all dimensions of the negotiation subject into the negotiation. Negotiation partners must avoid being blocked in positions and have to follow their interest, accepting adjustments of the former. Positions are specific demands/offers (for example, number of square meters per person, number of new houses to be built, *etc*.), while interests are desires or goals, things that people want to achieve in a conflict situation (such as improved housing conditions) [55]. Environmental equity represents the subject and the dimensions are the elements of the social, economic, and environmental impact categories (and they are analyzed in detail in Section 4.2). It should also be remembered that the final goal is not just to have an agreement or a policy (mutually accepted and beneficial), but to have it implemented. From this perspective, the use itself of the negotiation process, practiced with competence, dedication, and concern for overall satisfaction of interests, fosters relationships and stimulates partners to contribute to the implementation of the agreement [62].

### 4.2. Distributional Justice in Pata Rât

A sound body of information and knowledge is a prerequisite of paramount importance for the preparation and development of any negotiation, and, especially, for the implementation of agreements. In the present case, besides mastering negotiation skills and knowing the legal framework, deep understanding of the social, economic, and environmental reality is mandatory for a realistic, correct, and successful approach of EJ. The hereinafter thorough analysis of the social, economic, and environmental aspects are intended to support the quest for EJ in Pata Rât.

The environmental movements during the last decades imposed an environmental paradigm shift from the preservation of remote habitats to a more localized strategy on environmental improvements in the quality of life closer to the homes of affected humans [24]. For Central and Eastern Europe, four main patterns of the most common forms of environmental injustice were identified: exposure to hazardous waste and chemicals (settlements at contaminated sites); vulnerability to floods; differentiated access to potable water; and discriminatory waste management practices [36]. Within this framework, Pata Rât is affected by three of them: the first, the third, and the last form, while the second is not present, due to geographical and climatic characteristics of the area.

Pata Rât case is a conclusive example for the issue of environmental equity targeted to serve human justice, which becomes more evident in these cases where the elements of social justice co-exist with the main concern of the EJ movement [63], namely Roma people access to clean and healthy environment. The evident degradation of the environment in urban areas, affecting especially people from the periphery, has been materialized in a multitude of environmental, economic, and social public campaigns, which, unfortunately, remain with no result, turning into a new form of “eco-populism” [64], present, with priority, in electoral discourses. Therefore, the environmental movement, preoccupied mostly to clean up the environment, sometimes arrives to a point where it promotes practices that harm the environment itself [63] or the deprived social categories, in support of the privileged social class concerns, such as their own environment, jobs, logistics interest, or health care. In the case of Pata Rât, environmental concerns are viewed and analyzed as a determinant of the Roma minority living conditions, becoming a matter of life and death.

In general, the distributional justice deals with the role of class, race, gender, and ethnicity in the environmental degradation. Empirical evidence for environmental discrimination is obvious through the analysis of the following factors: A. social, B. environmental, and C. economic.

#### A. The Social Component of Environmental Equity

##### A.1. The Selected Population and Its Specific Problems

Population-specific problems in general, also recorded in the case of Roma minority from Pata Rât, occur when the life experience or behavior of a group results in their inequitable treatment [65]. It is about a group of people constantly pushed at the edge of the society, and here the “peripheral” concept must be understood both from geographical, territorial, point of view, and from an economic and social perspective [57]. Pata Rât is at the economic “periphery” and at the socially deprived outskirts of the agricultural and industrial productivity system of Cluj county [66]. It becomes a self-evident case of disproportionate burden, for which the county municipality followed, according to Bullard, the “path of least resistance” by locating this landfill in minority areas that are economically and politically powerless. Moreover, Roma minority has a long history of fighting environmental inequities: from climate-related working and living conditions up to those brought by industrialization, such as waste toxicity hazards or depletion of environmental services, generally concentrated in the geographic periphery of human settlements, like Pata Rât.

It is argued that there are two important characteristics of such population-specific problems. Firstly, they tend to be non-geographically specific [65]. This means that, generally, at country level, Roma people suffer from higher exposure to environmental risks than the rest of the population, because they are more likely to inhabit poorly maintained residences and deprived rural and urban areas (due to low incomes, cultural background, *etc*.). However, for the present study, the intensity of the environmental problems (judged in their larger social-economic-political context) faced by the Roma minority from Pata Rât stands out of the (non-geographically specific) ordinary level which is frequently encountered amongst the members of Roma population. This massive exposure is limited to Pata Rât location and cannot be extrapolated to the entire Cluj county, nor nationwide, transforming it into a geographically specific one for Pata Rât. The extent of the mix of social-economic-environmental impacts generated by Pata Rât landfill is both devastating and unique, bearing a strong Roma stigmatization imprint. Secondly, these characteristics are created by the population’s lifestyle specificity; in this case, the nomadism with the related habits can be mentioned. These lifestyle characteristics may be rooted and encapsulated in a particular historical-political national (Romanian) background. We are dealing at Pata Rât with more than 2000 Roma people, most of the time voiceless in environmental and economic-social debates related to their future. In fact, it is common among those subjected to harm to have little history of civic and political participation [2]. In some cases, as Jamieson observes, participation is denied for other reasons than institutional or regulatory framework failure [67]. It could be a path-dependency of this community of not acting, not disturbing, of remaining invisible, rooted in a historical, not so remote, past. The documentarily verified presence of the Roma on the actual territory of Romania dates back to 1385 [68], and they suffered one of the longest episodes of slavery in human history (471 years, between 1385–1856) [69]. Extermination in camps in Eastern Europe, the Balkans or the former Soviet Union did not have the dimensions of Auschwitz, but it was organized on the same ideological premises, using similar bureaucratic plans, industrial methods, and racial marks. Unfortunately, the scant nature of studies made it impossible to know the exactly number of deportees, ranging from 25,000 to 300,000 [69]. The widespread institutional and social stereotyping of the “țigan” (Roma) as the lowest social category, which had to be enslaved, even if they played an important economic role in society, served to create a common group within the social hierarchy against which all other social and ethnic groups in the Romanian principalities could considered themselves as superior, based on different attributes such as free, white, or Christian [70]. It can be claimed to some extent that a political failure—incapability to act and change this secular way of non-action—can be incriminated. This ethnic group could be blamed only from the perspective of choosing the easiest way by not participating in social governance, that of having poverty as a “handicap”, or that of cringing under society’s mentality. However, this behavior is justified by the fact that we deal with an omnipresent preconceived scenario, from the justice to the street, in which, regardless of their nature, Roma people are perceived as the subjects of incrimination. This minority, according to national Romanian normativity, is not legally denied from a fair share and treatment, but they are still absent from the community governance, over which justice is supposed to prevail [67]. Studies of the Centre for Public Policy Studies from Romania, mentioned by Petcuț, reveal a contemporary racism on Roma minority at community level [69]. Almost 36% of respondents consider that this ethnic group should be forced to live separately from the rest of society, 31% say that there should be pubs and shops where their access should not be allowed, and 48% mention that the state should take action on birth control with respect to them. In other Member States, the massive “exports” from Eastern Europe after the last EU enlargements, due to the free movement liberties and social rights, contributed to negative stereotyping of Roma people. As a result, in several Member States majorities express unfavorable views of Roma (Italy 85%, France 66%, and United Kingdom 50%) [71]. In most of the cases, Roma’s poor economic and environmental life chances, dysfunctional social relations, or social exclusion were rooted, by the respondents, in Romas’ dysfunctional behavior.

##### A.2. Social Consequences of Living with Environmental Problems at the Pata Rât Landfill

Three factors cumulate to tailor the bleak destiny of Pata Rât residents: the fact they are a minority (thus having a lower power that the majority), their ethnicity—Roma (which was historically oppressed and repelled), and the fact that they are living on and off what society rejects and dumps—the landfill. In the Romanian context, being of Roma ethnicity and being a minority is not the same problem. The membership to Roma minority makes a huge difference in the way people are perceived and treated: they are seen differently from other minorities (negatively), in contrast with positive perceptions related to Hungarian, German, Serbian or other minorities living in this country. This is the reason why it was necessary to individualize this factor, being Roma, with a strong impact on their existence, besides the other two: belonging to a minority and living in a landfill. If we studied other minorities in Romania, the use of “ethnic minority” as a single factor would be the right one. The previously mentioned trio leads to a significant overlapping (in the mentality of the outside Pata Rât community) of these Roma residents’ identity with the idea of waste, repulse, and avoidance. For the present analysis, the environmental aspect—the pollution—fosters the social inequalities for Roma citizens.

Social security, through its redistributive character, has a major role in poverty reduction and alleviation, preventing exclusion and promoting social inclusion [72]. The right to social security contains the right not to be the subject of restrictions regarding existing social security coverage, as well as the right to equal enjoyment of adequate protection from social risks. In Pata Rât, the availability of social security scheme or the accessibility, including affordability to it, are entirely missing, therefore, Pata Rât residents are unable to benefit from adequate social security. Access to education and health care systems has also been negatively impacted. On several occasions, ambulances refused to respond to calls from Pata Rât, and even when they did, the timeframe was between 2.5 and 3 h for an ambulance to arrive (from a distance of about 18 km, where the city center is located). Many children (25%) reported being subjected to racist remarks at school, while 10% of children were placed in special education classes since they were relocated to Pata Rât, despite having never previously been in such educational structures [73]. Based on European Roma Rights Centre data (2012) the average living space in Pata Rât is 4 m^2^, one bathroom is shared between at least 17 people, there is no water connection in any of the modular homes, and 89% of interviews report that they do not have cooking facilities; 30% of residents declare some physical illness since the eviction, an increase of 14%; 51% of residents do not have a national ID for their new address after eviction and displacement to Pata Rât [73]. Therefore, restrictions on access to social security schemes are present *de facto*, even if *de jure* a legal framework of protection, comprising the obligation to respect, to protect, and to fulfill Roma minority rights, exists. The gap between normativity and reality is drifting on the state’s failure to take sufficient and appropriate action to achieve the right to social security, a failure caused by Roma stigma, and by the status of landfill resident.

#### B. Environmental Related Component

Environmental injustice can be historical and present/future [65]. Pata Rât incorporates both features: it is a case of historical environmental injustice, resulted from decisions of Cluj-Napoca municipality to deposit there the city waste (for decades until 2015), which still has effects in present communities, due to cumulative past exposure to contamination. At the same time, we deal with a present and future environmental injustice through the present lack of decontamination measures, which endanger the residents. The landfill must be secured and monitored in the post-closure phase, to prevent further pollution, because the biochemical and physical processes in the deposit were not fully stabilized at the moment of closing the landfill [47].The future dimension of environmental equity has a high probability to extend in time as a result of the high financial and physical efforts required for decontamination. At the storage platform from Pata Rât (that covers 18 hectares), the existence of an additional amount of 541,835 m^3^ of waste compared to the one mentioned in the tender documentation was found and the value of additional works involving neutralizing and greening amounted to 1,775,933 Euros [74]. Unfortunately, in Romania, storage is still the primary option of municipal waste disposal and only 5% of domestic waste is recycled [75]. The country faces serious impediments in shifting from a traditional waste management system based on landfilling to a sustainable and integrated system [76].

The historic nature of Pata Rât pollution causes dramatic alteration of environmental components, including human health: air pollution by wind-blown suspensions, water, and soil contamination with nitrites, nitrates, or heavy metals (Cr, Cd, Cu, Ni, Pb, Zn), landscape modifications, and biodiversity loss [77,78]. Surface water is the most affected environmental component at Pata Rât, being degraded and improper for life, and, thus, requiring control and prevention measures against the pollution of surface water with leachate; leachate is highly loaded with chlorides, sulfates, ammonia, and petroleum ether extractable substances, and it exceeds the limit value for Cu, Ni, and total P and nitrates [46]. Methane and carbon dioxide have significant contributions to climate change and MSW landfills, in general, are considered as an important source of anthropogenic methane emissions. Carbon dioxide and methane emission performed with *in situ* measurements by using the closed chamber method revealed high values, with eleven times lower emission of methane than carbon dioxide, possibly as a consequence of: the composition of the solid waste, the uncontrolled leaching of organic matter, the open burning of solid waste, and climatic conditions [46]. Soil sample analysis indicated contamination values far beyond the accepted limits: Pb was 38.6 mg/kg d.m. (dry material) (normal value: 20 mg/kg d.m.), Se was 8.2 mg/kg d.m. (normal value: 1 mg/kg d.m.), Cu was 103.5 mg/kg d.m. (normal value: 20 mg/kg d.m.), Mn was 11,398.4 mg/kg d.m. (normal value: 900 mg/kg d.m.), Ba was 338.4 mg/kg d.m. (normal value: 150 mg/kg d.m.) [79].

**Figure 3:**
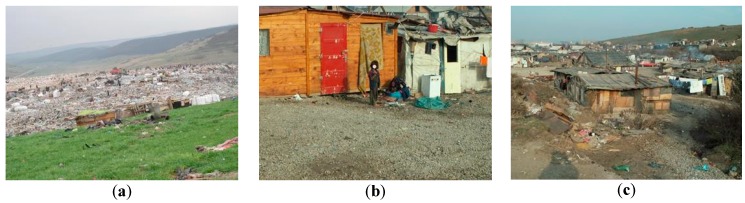
Life on the Pata Rât waste dump: (**a**), (**b**), (**c**) (photos: Ciprian Bodea).

Historical and present/future environmental contamination at Pata Rât dumping site creates improper life conditions for the residents (Figure 3), and infringes both national and international regulations. Thus, the Romanian Constitution guarantees, through Article 35—The right to a healthy environment: “(1) The State recognizes the right of everyone to a healthy and ecologically balanced environment; (2) The State shall provide the legislative framework for exercising this right; (3) Natural and legal persons have the duty to protect and improve the environment” [80]. Moreover, Article 135 (letter e) imposes on the State the obligation to secure the “environmental protection and recovery, as well as preservation of the ecological balance” [80]. Article 3 of the Treaty on European Union (TEU) states that “the Union shall establish an internal market. It shall work for the sustainable development of Europe based on (…) a high level of protection and improvement of the quality of the environment” and the foster of the environmental development is a primary aim of eradicating poverty (Article 21 TEU) [81]. General Comment No. 4 of the Committee on Economic, Social and Cultural Rights declares that “housing should not be built on polluted sites nor in immediate proximity to pollution sources that threaten the right to health of the inhabitants” [82].

#### C. Economic Well-Being

All problems faced in the context the lack of EJ have an economic component [65]. In general, a low level of environmental quality factors, as is the case of Pata Rât, is associated with economically depressed populations [83]. Environmental inequity is both a consequence and a source of economic impediments—it is rooted in and generates pecuniary disadvantages. Thus, communities under economic stress choose to trade their environmental benefits (water quality, biodiversity services, aesthetic values, *etc*.) for economic gains or do not have the means to detect, alleviate, and protect themselves against contamination; also, people who suffer from environmental inequity face a higher risk to worsen their economic status. Environmental equity assessment in the case of Roma people’s pecuniary needs must gravitate around measuring the risk exposure differences between the under-analysis population and rest of Cluj inhabitants, taking into account if they had complete and correct information about the risks they suffered and how much freedom of choice the population actually had in making a decision [65]. Lack of constant income, being evicted, jobless, or without property, depict their precarious material situation (Table 1), which, along with social and environmental hardships, prevent them to escape from their economic vulnerability. The endemic anti-Roma discrimination and prejudicial attitudes within majority non-Roma populations represent a significant obstacle, inhibiting Roma’s inclusion in the paid labor market [84].

The only benefit this socially vulnerable people inhabiting Pata Rât hazardous location have is a short-term economic one, namely, the income they obtain from sorting the dumped municipal waste. This is, in fact, an economic (as it is performed for a lower monetary compensation) and environmental (as selection turns into recycling) service they deliver to the waste generating community. It is worth mentioning that Pata Rât Roma residents represent nowadays the only current solution for garbage selection, which should increase the value of Pata Rât community for Cluj inhabitants: through their valorization in the collective mental and through an equitable financial reward. A study of Pop *et al.*, estimates that 50% of the total generated waste has the potential to be used for energy recovery and more than 37% could be recycled. Approximately 793 permanent garbage pickers recover around 12,230 tons annually, while 2366 street pickers collect 4600 tons of recyclable materials per year, and 400 itinerant waste collectors mostly collect metals, which, at county level, means that 8% of the total municipal waste is recovered by the informal sector, while only 5% through the formal sector [51].

A key factor in achieving a paradigm shift in perception of this minority and in improving their life conditions is the promotion of the idea of waste as a source of income. Transforming waste to income and Roma people to citizens with stable jobs with equitable pay has at least the following economic benefits: recycling associated ones (waste pollution reduction, use of new raw-material sources, *etc*.), production and commercialization/on-site use of bio-gas, reduction of costs associated with health problems, with justice (lower law infringements, with consequent procedural and detention costs), social care (unemployment, low-income compensations), and costs related to reduction and elimination of at-source pollution. This aspiration is achievable, in terms of financial allocations, due to the high amount of money assigned for the 2014–2020 period: Romania will receive 22.9 billion Euros in EU funds, out of which 15.95 billion Euros will come from the European Social Fund (ESF). Of this amount, at least 30.8% will be spent on the ESF, with a minimum of 20% of that going towards the promotion of social inclusion and combating poverty, especially through financing Roma-related measures [85].

## 5. Conclusions

In the frame of environmental equity, the Pata Rât MSW landfill is generator of environmental degradation, which causes disproportionate social, economic, and environmental impacts. The present paper advocates for the necessity to bring environmental equity within Pata Rât Roma community, relying on the answers to three questions: “Does environmental equity exist in Pata Rât Roma community?”, “How can it be attained/ improved?”, and “To what extent can it be brought to targeted people?”.

The fundamental principle in designing the current research was that equity means that similarly situated people should be treated equally, regardless of the connotations [86] and of people’s characteristics (gender, ethnicity, color, religion, share in total population, *etc.*). The marginalization of Pata Rât Roma community is multifaceted, being rooted in and giving birth to social, economic, and environmental huge disparities. Consequently, environmental equity does not exist for Roma minority.

In the present context, in order to answer to the second question, the negotiation is the endorsed means to achieve environmental equity. The reasoning behind this choice is that negotiation can manage, under certain conditions, such as segregation, discrimination, and avoidance, an important part of conflict situations (like those between the majority and the Roma minority) that the market and the soft and hard law instruments are not capable or do not want to solve (or they do it, but at higher costs or ineffectively). Therefore, the negotiation will represent a strategy for adaptation and equilibrium, a smart resource that can be used with better results and with lower costs than other methods to allocate and distribute resources, to efficiently integrate interests and preferences relatively inconsistent with each other, to extinguish the conflict in a cooperative manner and to create net value from the parts’ original positions [87]. Negotiation is supported also due to its capacity to implement the principle of proximity, which aims to encourage the local communities in assuming responsibilities for taking decisions as close as possible to their interests.

The analysis provides meaningful and qualitative evidence that evaluation of environmental equity is largely subjective, because of its complexity and specificity related to geographical, historical, cultural characteristics, and political interests, and therefore, the extent to which it can be brought within Pata Rât Roma community may be judged (based on stakeholders’ perceptions) in “shades of grey”, not only in black and white (not at all or totally achieved in relation to its distributional and procedural components, under the “guard” of equity, recognition, and participation). This nuanced approach, its awareness and valorization is a key element on the long way to acceptance and change, so that, an environmentally just and equitable outcome for the Roma people of the Pata Rat landfill can be attained. Therefore, instead of giving quantitative results, a qualitative one was obtained, which indicated an order of preference—degree of reaching environmental equity. To advance in finding an answer to the third question, a list of criteria was designed, derived from sustainable development concept (focused on economic, social, and environmental dimensions) and from Maslow’s hierarchy of needs. According to stakeholders’ judgements, using the evaluation matrix, the preferred action turned out to be the maintenance of the Roma population on-site, improving their living conditions through public intervention. However, it should be remembered that the results obtained through the evaluation matrix are not representative for the whole groups of stakeholder and, therefore, the research should be extended to a larger population in the future. Also, the criteria could be adjusted according to the particular visions of the stakeholders that will be involved in the real negotiation that will pursue the achievement of EJ.

The findings of the research can serve to guide policy-makers and private entities as a starting point in designing interventions to alleviate the hardships generated by discrimination, racism, economic vulnerabilities, and the blighted Pata Rât landfill environment, that continue to negatively stereotype Roma communities into a peripheral position in society and which impede the minority to benefit from environmental equity.

## Figures and Tables

**Table 1:** Economic situation of Pata Rât residents (in 2012 and 2014) [43,44].

**Employment Status (Multiple Options; Aged 18–64) (UNDP 2012)**	**Percentage of Pata Rât Total Population**
Employed, With Contract	9.4%
Self-Employed	1.2%
Employer	0.4%
Occasional Worker, With or Without a Contract	14.2%
Worker on the Garbage Dump	44.7%
Registered Unemployed	1.2%
Pensioner	5.6%
Total Income * Per Household Members (UNDP 2012)	**Number of Households**
200 and Above Euros/Month	8
156–199 Euros/Month	7
111–155 Euros/Month	27
67–110 Euros/Month	45
Below 66 Euros/Month	159
**Economic Autonomy (UNDP 2014)**	**Number of Households**
Own a House With Property Documents in Another Area	33
Own a House Without Having Property Documents	13
Own Both Land for Agriculture and a House	7
Family Considered Economically Autonomous **	20
Most Economically Vulnerable Families ***	145

* Exchange rate: 1 euro = 4.5 Romanian Lei; ** Criteria of economic autonomy are: obtaining income outside of the landfill and possessing at least one of the three economic resources (Internet, electric meter, or car); *** Families which do not obtain income outside of the landfill and do not have any of the resources mentioned for economic autonomy.

**Table 2:** Criteria evaluation (their performance level within an action option) and importance.

Criteria	Criteria Evaluations: Average Scores of All Stakeholders per Criterion for Each Action Option			Importance of Each Criterion Given by Each Group of Stakeholders *				Average Importance of Each Criterion (Resulted From All Stakeholders) for Each Criterion
	On-Site	Off-Site	“Do Nothing”	Local Authorities	Non-Roma City Residents	NGOs	Pata Rât Residents	
**A. Social Perspective**								
1. Physiological								
They ** are able to cover their physiological needs (food, drink, *etc*.)	4.25	4.76	2.67	10	10	10	10	**10.00**
2. Safety needs								
They will have secure housing	8.17	8.17	1.75	9.7	9	10	10	**9.67**
They are safe from abuse from their neighbors and from the people from the city	8.08	6.17	5.42	7.7	4.67	9.33	10	**7.92**
They are a threat to the population of the city—through the crimes they commit ***	8.25	5.17	5.17	9	10	9.67	6	**8.67**
They are a threat to the health of the population of the city—through their lack of hygiene, diseases, and bad habits ***	6.58	4.42	2.92	9.7	10	7.67	1	**7.08**
They will have access to education	8.25	6.08	1.58	10	8.33	10	9	**9.33**
They will have access to employment	8.08	5.58	1.67	10	10	10	7.667	**9.42**
They will have access to health care	8.00	5.67	1.83	10	9	10	10	**9.75**
3. Belonging								
They are well integrated in and accepted by the community where they live	9.83	5.08	7.33	10	9	10	9	**9.50**
4. Respect from others								
They are treated as equals by the people from their community	9.33	5.67	6.58	5.7	2.67	8.67	8.667	**6.42**
They are treated as equals by the people outside their community	7.17	4.33	1.92	5.7	1	8.67	8.667	**6.00**
5. Self-actualization								
They have the opportunity and capacity to improve their lives and become fulfilled as a human being	7.17	5.00	1.75	7.3	6.33	10	7.667	**7.83**
***B. Economic perspective***								
They contribute to the local economy (through the work they perform)	8.67	5.50	4.33	10	9	10	6	**8.75**
They generate costs with allowances, damages they may produce, *etc*. ***	7.00	3.58	2.00	10	9.33	10	2.333	**7.92**
**C. *Environmental perspective***								
The environmental pollution threatens their lives ***	6.00	8.25	1.42	8.7	5.67	10	9	**8.33**
They don’t contribute any longer to environment protection through waste selection	6.67	2.92	5.42	7.7	1.67	5.33	1	**3.92**
Average score for assigned performance of each criterion for each action option ****	**7.59**	**5.39**	**3.36**					
Weighted average score for each action option *****	**994.52**	**721.60**	**424.22**					

* The scores indicate the stakeholders group average, based on the evaluation/importance assigned by the three representatives of each group of stakeholders; ** “They” means Pata Rât residents from the village of shacks; *** Reverse coding (used for the results included here compared to the original evaluations/codes presented to people); **** The results were calculated as follows: (4.25 + 8.17 + …. + 6.67)/16 = 7.59; (4.76 + 8.17 + … + 2.92)/16 = 5.39; (2.67 + 1.75 + … + 5.42)/16 = 3.36; ***** The results were calculated as follows: 4.25 × 10 + 8.17 × 9.67 + …. + 6.67 × 3.92 = 994.52; 4.76 × 10 + 8.17 × 9.67 + … + 2.92 × 3.92 = 721.60; 2.67 × 10 + 1.75 × 9.67 + … + 5.42 × 3.92 = 424.22.

## Abbreviations

The following abbreviations are used in this manuscript:

d.m.	dry material
ECHR	European Court of Human Rights
EJ	Environmental Justice
EU	European Union
ID	Identification Document
MSW	Municipal Solid Waste
NGO	Non-Governmental Organization
ESF	European Social Fund
TEU	Treaty on European Union
